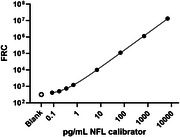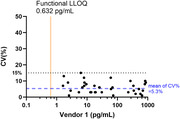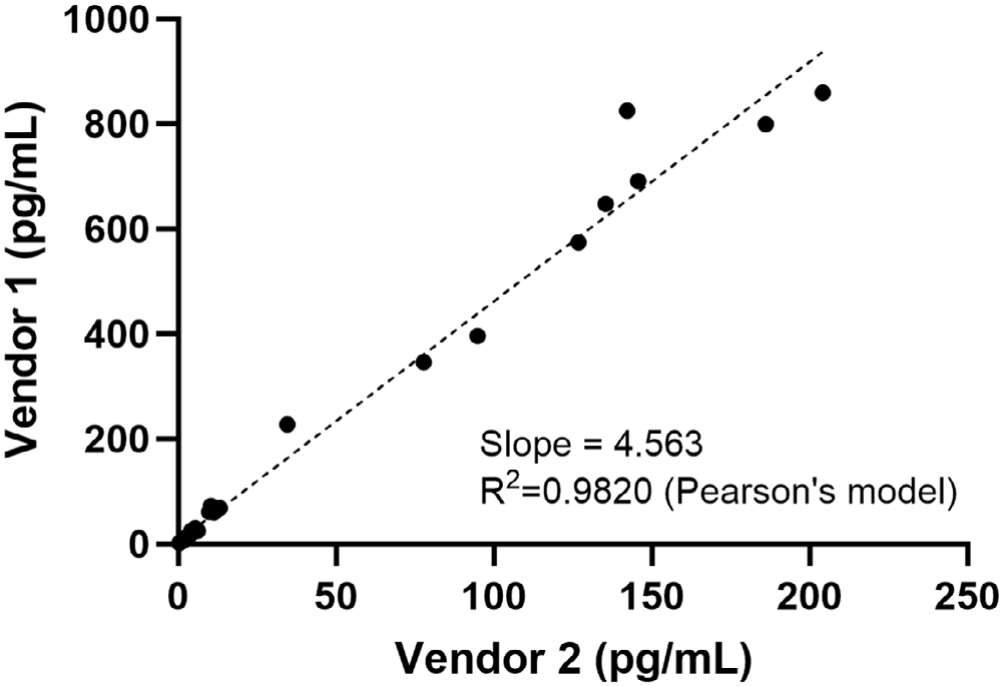# A scalable solution for precisely measuring low abundant neurobiomarkers from micro‐sampling

**DOI:** 10.1002/alz.093328

**Published:** 2025-01-09

**Authors:** Tsz Wing Fan, WonHee Kim, Corinne Thomas, Wenjing Jiang, Jeremy Tan, Feng Xuan

**Affiliations:** ^1^ SPEAR BIO INC, Woburn, MA USA

## Abstract

**Background:**

Cutting‐edge ultrasensitive immunoassay platforms have unveiled the potential of blood‐based biomarkers, offering detection at low fg/mL levels for early neurodegenerative disorder prognosis, screening, and therapeutic monitoring. Current immunoassays, such as single molecule array (SIMOA) and mesoscale multi‐array (MSD), face limited adoption due to their reliance on specialized equipment. Additionally, they require immobilization of probe reagents and a washing process, demanding tens of thousands of proteins to achieve the Limit of Detection (LOD), leading to the requirement of high sample volume and high affinity antibodies for fg/mL sensitivity. Addressing the escalating need for non‐invasive, small‐volume sample collection, along with an accessible platform for frequent longitudinal monitoring and population‐scale screening, a solution with these features is imperative.

**Method:**

Introducing Successive Proximity Extension Amplification Reactions (SPEAR), which employs a unique two‐factor authentication mechanism that eliminates the background in conventional homogeneous assay, detects down to single‐digit copies of proteins. SPEAR utilizes a simple three‐step wash‐free workflow adaptable to any liquid handler and readable on ubiquitous PCR machines. To showcase its utility in detecting neurobiomarkers at low concentrations in blood, we demonstrate NFL assay using just 1‐mircorliter diluted plasma sample to reach low fg/mL sensitivity with excellent precision. Comprehensive testing included spike‐in recovery, dilution linearity and specificity against neurofilaments (Nf‐M, Nf‐H, peripherin, and α‐internexin), precision and accuracy.

**Result:**

SPEAR NFL assay exhibited remarkable LOD and Lower Limit of Quantification (LLOQ) from two commercial readily available NFL antibody pairs. The LOD was measured at 51 and 90 fg/mL using 1 µL of 1:4 diluted plasma. Robust precision was indicated by low intraplate and lot‐to‐lot %CV (6% and 7%, respectively), and high concordance against Simoa® NF‐light™ V2 Advantage Kit and MSD Splex kit using 36 K2EDTA plasma samples.

**Conclusion:**

SPEAR has demonstrated validated sensitivity against existing NFL detection platforms. Its standout features, including minimal sample volume requirement and compatibility with standard PCR machines, coupled with advances in portable PCR machines and remote collection devices, position SPEAR as a robust and accessible tool for neurodisease diagnostic and research.